# Chemistry and Biological Activities of Flavonoids: An Overview

**DOI:** 10.1155/2013/162750

**Published:** 2013-12-29

**Authors:** Shashank Kumar, Abhay K. Pandey

**Affiliations:** Department of Biochemistry, University of Allahabad, Allahabad 211002, India

## Abstract

There has been increasing interest in the research on flavonoids from plant sources because of their versatile health benefits reported in various epidemiological studies. Since flavonoids are directly associated with human dietary ingredients and health, there is need to evaluate structure and function relationship. The bioavailability, metabolism, and biological activity of flavonoids depend upon the configuration, total number of hydroxyl groups, and substitution of functional groups about their nuclear structure. Fruits and vegetables are the main dietary sources of flavonoids for humans, along with tea and wine. Most recent researches have focused on the health aspects of flavonoids for humans. Many flavonoids are shown to have antioxidative activity, free radical scavenging capacity, coronary heart disease prevention, hepatoprotective, anti-inflammatory, and anticancer activities, while some flavonoids exhibit potential antiviral activities. In plant systems, flavonoids help in combating oxidative stress and act as growth regulators. For pharmaceutical purposes cost-effective bulk production of different types of flavonoids has been made possible with the help of microbial biotechnology. This review highlights the structural features of flavonoids, their beneficial roles in human health, and significance in plants as well as their microbial production.

## 1. Introduction

Flavonoids consist of a large group of polyphenolic compounds having a benzo-**γ**-pyrone structure and are ubiquitously present in plants. They are synthesized by phenylpropanoid pathway. Available reports tend to show that secondary metabolites of phenolic nature including flavonoids are responsible for the variety of pharmacological activities [1, 2]. Flavonoids are hydroxylated phenolic substances and are known to be synthesized by plants in response to microbial infection [3]. Their activities are structure dependent. The chemical nature of flavonoids depends on their structural class, degree of hydroxylation, other substitutions and conjugations, and degree of polymerization [4]. Recent interest in these substances has been stimulated by the potential health benefits arising from the antioxidant activities of these polyphenolic compounds. Functional hydroxyl groups in flavonoids mediate their antioxidant effects by scavenging free radicals and/or by chelating metal ions [5, 6]. The chelation of metals could be crucial in the prevention of radical generation which damage target biomolecules [7, 8]. As a dietary component, flavonoids are thought to have health-promoting properties due to their high antioxidant capacity both *in vivo* and *in vitro* systems [9, 10]. Flavonoids have ability to induce human protective enzyme systems. The number of studies has suggested protective effects of flavonoids against many infectious (bacterial and viral diseases) and degenerative diseases such as cardiovascular diseases, cancers, and other age-related diseases [2, 9, 10]. The mechanisms involved in protection provided by flavonoids are described separately in this review. Flavonoids also act as a secondary antioxidant defense system in plant tissues exposed to different abiotic and biotic stresses. Flavonoids are located in the nucleus of mesophyll cells and within centers of ROS generation. They also regulate growth factors in plants such as auxin [11]. Biosynthetic genes have been assembled in several bacteria and fungi for enhanced production of flavonoids [12]. This review deals with the structural aspects of flavonoids and their protective roles against many human diseases. Functions of flavonoids in plants and their microbial production have also been described.

## 2. Chemistry of Flavonoids

Flavonoids are a group of natural compounds with variable phenolic structures and are found in plants. In 1930 a new substance was isolated from oranges. At that time it was believed to be a member of a new class of vitamins and was designated as vitamin P. Later on it became clear that this substance was a flavonoid (rutin) and till now more than 4000 varieties of flavonoids have been identified [13].

**Figure 1:**
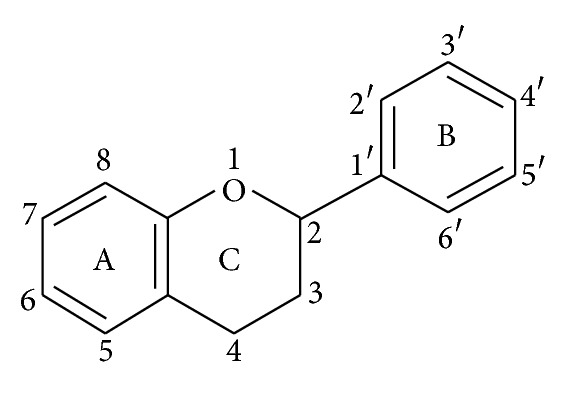
Basic flavonoid structure.

Chemically flavonoids are based upon a fifteen-carbon skeleton consisting of two benzene rings (A and B as shown in Figure 1) linked via a heterocyclic pyrane ring (C). They can be divided into a variety of classes such as flavones (e.g., flavone, apigenin, and luteolin), flavonols (e.g., quercetin, kaempferol, myricetin, and fisetin), flavanones (e.g., flavanone, hesperetin, and naringenin), and others. Their general structures are shown in Table 1. The various classes of flavonoids differ in the level of oxidation and pattern of substitution of the C ring, while individual compounds within a class differ in the pattern of substitution of the A and B rings [13].

Flavonoids occur as aglycones, glycosides, and methylated derivatives. The basic flavonoid structure is aglycone (Figure 1). Six-member ring condensed with the benzene ring is either a *α*-pyrone (flavonols and flavanones) or its dihydroderivative (flavonols and flavanones). The position of the benzenoid substituent divides the flavonoid class into flavonoids (2-position) and isoflavonoids (3-position). Flavonols differ from flavanones by hydroxyl group at the 3-position and a C2–C3 double bond [40]. Flavonoids are often hydroxylated in positions 3, 5, 7, 2, 3′, 4′, and 5′. Methyl ethers and acetyl esters of the alcohol group are known to occur in nature. When glycosides are formed, the glycosidic linkage is normally located in positions 3 or 7 and the carbohydrate can be L-rhamnose, D-glucose, glucorhamnose, galactose, or arabinose [41].

### 2.1. Spectral Characteristics of Flavonoids

Studies on flavonoids by spectroscopy have revealed that most flavones and flavonols exhibit two major absorption bands: Band I (320–385 nm) represents the B ring absorption, while Band II (250–285 nm) corresponds to the A ring absorption. Functional groups attached to the flavonoid skeleton may cause a shift in absorption such as from 367 nm in kaempferol (3,5,7,4′-hydroxyl groups) to 371 nm in quercetin (3,5,7,3′,4′-hydroxyl groups) and to 374 nm in myricetin (3,5,7,3′,4′,5′-hydroxyl groups) [42]. The absence of a 3-hydroxyl group in flavones distinguishes them from flavonols. Flavanones have a saturated heterocyclic C ring, with no conjugation between the A and B rings, as determined by their UV spectral characteristics [43]. Flavanones exhibit a very strong Band II absorption maximum between 270 and 295 nm, namely, 288 nm (naringenin) and 285 nm (taxifolin), and only a shoulder for Band I at 326 and 327 nm. Band II appears as one peak (270 nm) in compounds with a monosubstituted B ring, but as two peaks or one peak (258 nm) with a shoulder (272 nm) when a di-, tri-, or *o*-substituted B ring is present. As anthocyanins show distinctive Band I peak in the 450–560 nm region due to hydroxyl cinnamoyl system of the B ring and Band II peaks in the 240–280 nm region due to the benzoyl system of the A ring, the colour of the anthocyanins varies with the number and position of the hydroxyl groups [44].

## 3. Flavonoid Rich Food and Medicinal Plants

Flavonoids are the most common and widely distributed group of plant phenolic compounds, occurring virtually in all plant parts, particularly the photosynthesising plant cells. They are a major coloring component of flowering plants. Flavonoids are an integral part of human and animal diet. Some food sources containing different classes of flavonoids are given in Table 2. Being phytochemicals, flavonoids cannot be synthesized by humans and animals [45]. Thus flavonoids found in animals are of plant origin rather than being biosynthesized in situ. Flavonols are the most abundant flavonoids in foods. Flavonoids in food are generally responsible for colour, taste, prevention of fat oxidation, and protection of vitamins and enzymes [46]. Flavonoids found in the highest amounts in the human diet include the soy isoflavones, flavonols, and the flavones. Although most fruits and some legumes contain catechins, the levels vary from 4.5 to 610 mg/kg [47]. Preparation and processing of food may decrease flavonoid levels depending on the methods used. For example, in a recent study, orange juices were found to contain 81–200 mg/L soluble flavanones, while the content in the cloud was 206–644 mg/L which suggest that the flavanones are concentrated in the cloud during processing and storage [48]. Accurate estimation of the average dietary intake of flavonoids is difficult, because of the wide varieties of available flavonoids and the extensive distribution in various plants and also the diverse consumption in humans [49].

Recently there has been an upsurge of interest in the therapeutic potential of medicinal plants which might be due to their phenolic compounds, specifically to flavonoids [50, 51]. Flavonoids have been consumed by humans since the advent of human life on earth, that is, for about 4 million years. They have extensive biological properties that promote human health and help reduce the risk of diseases. Oxidative modification of LDL cholesterol is thought to play a key role during atherosclerosis. The isoflavan glabridin, a major polyphenolic compound found in *Glycyrrhiza glabra *(Fabaceae), inhibits LDL oxidation via a mechanism involving scavenging of free radicals [52]. Several epidemiologic studies have suggested that drinking either green or black tea may lower blood cholesterol concentrations and blood pressure, thereby providing some protection against cardiovascular disease. Flavonoids are also known to influence the quality and stability of foods by acting as flavorants, colorants, and antioxidants [53, 54]. Flavonoids contained in berries may have a positive effect against Parkinson's disease and may help to improve memory in elderly people. Antihypertensive effect has been observed in total flavonoid fraction of *Astragalus complanatus *in hypertensive rats [55]. Intake of antioxidant flavonoids has been inversely related to the risk of incidence of dementia [56].  Table 3 summarizes some of the medicinal plants rich in flavonoid contents.

Solubility may play major role in the therapeutic efficacy of flavonoids. Low solubility of flavonoid aglycones in water coupled with its short residence time in the intestine as well as its lower absorption does not allow humans to suffer acute toxic effects from the consumption of flavonoids, with the exception of a rare occurrence of allergy. The low solubility of the flavonoids in water often presents a problem for its medicinal applications. Hence, the development of semisynthetic, water-soluble flavonoids, for example, hydroxyethylrutosides and inositol-2-phosphatequercetin, has been implicated for the treatment of hypertension and microbleeding [57].

## 4. Metabolism of Flavonoids in Humans

**Figure 2:**
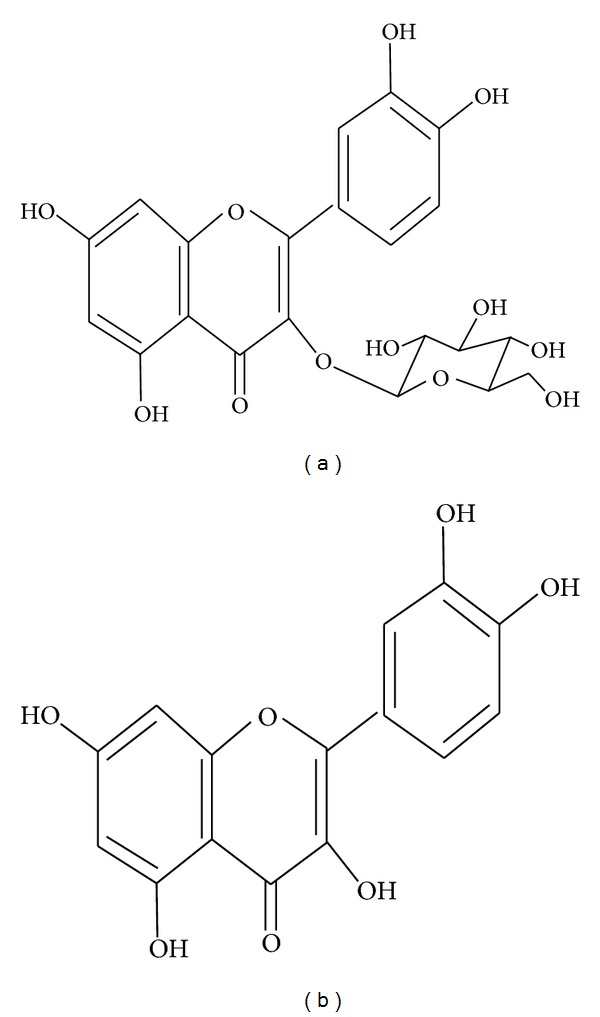
Structure of (a) flavonoid glycoside and (b) aglycone flavonoid.

The absorption of the dietary flavonoids liberated from the food by chewing will depend on its physicochemical properties such as molecular size, configuration, lipophilicity, solubility, and pKa. The flavonoid can be absorbed from the small intestine or has to go to the colon before absorption. It may depend upon structure of flavonoid, that is, whether it is glycoside or aglycone. Most flavonoids, except for the subclass of catechins, are present in plants bound to sugars as *b*-glycosides (Figure 2). Aglycans can be easily absorbed by the small intestine, while flavonoid glycosides have to be converted into aglycan form [58].

The hydrophilic flavonoid glucoside such as quercetin are transported across the small intestine by the intestinal Na^+^-dependent glucose cotransporter (SGLT1) [58]. An alternative mechanism suggests that flavonoid glucosides are hydrolyzed by lactase phloridzin hydrolase (LPH), a *β*-glucosidase on the outside of the brush border membrane of the small intestine. Subsequently, the liberated aglycone can be absorbed across the small intestine [59]. The substrate specificity of this LPH enzyme varies significantly in a broad range of glycosides (glucosides, galactosides, arabinosides, xylosides, and rhamnosides) of flavonoids [60]. The glycosides which are not substrates for these enzymes are transported toward the colon where bacteria have ability to hydrolyze flavonoid glycosides, but simultaneously they will also degrade the liberated flavonoid aglycones [61]. Since absorption capacity of the colon is far less than that of the small intestine, only trivial absorption of these glycosides is to be expected.

**Figure 3:**
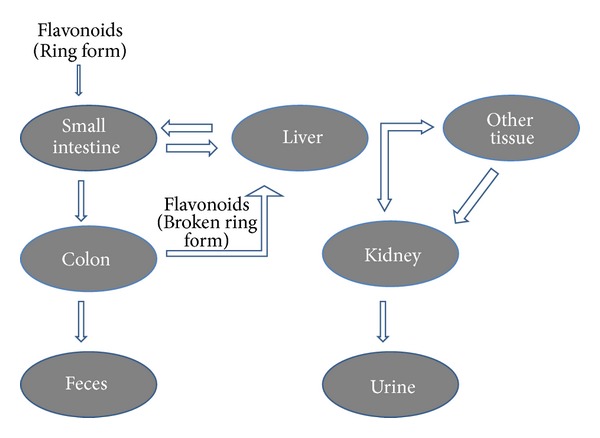
Compartments involved in the metabolism of flavonoid.

After absorption, the flavonoids are conjugated in the liver by glucuronidation, sulfation, or methylation or metabolized to smaller phenolic compounds [62]. Due to these conjugation reactions, no free flavonoid aglycones can be found in plasma or urine, except for catechins [63]. Depending on the food source bioavailability of certain flavonoids differs markedly; for example, the absorption of quercetin from onions is fourfold greater than that from apple or tea [64]. The flavonoids secreted with bile in intestine and those that cannot be absorbed from the small intestine are degraded in the colon by intestinal microflora which also break down the flavonoid ring structure (Figure 3). Oligomeric flavonoids may be hydrolyzed to monomers and dimers under influence of acidic conditions in the stomach. Larger molecules reach the colon where they are degraded by bacteria. The sugar moiety of flavonoid glycosides is an important determinant of their bioavailability. Dimerization has been shown to reduce bioavailability. Among all the subclasses of flavonoids, isoflavones exhibit the highest bioavailability [65]. After ingestion of green tea, flavonoid content is absorbed rapidly as shown by their elevated levels in plasma and urine. They enter the systemic circulation soon after ingestion and cause a significant increase in plasma antioxidant status [66].

## 5. Biological Activities of Flavonoids

### 5.1. Antioxidant Activity

Flavonoids possess many biochemical properties, but the best described property of almost every group of flavonoids is their capacity to act as antioxidants. The antioxidant activity of flavonoids depends upon the arrangement of functional groups about the nuclear structure. The configuration, substitution, and total number of hydroxyl groups substantially influence several mechanisms of antioxidant activity such as radical scavenging and metal ion chelation ability [4, 67]. The B ring hydroxyl configuration is the most significant determinant of scavenging of ROS and RNS because it donates hydrogen and an electron to hydroxyl, peroxyl, and peroxynitrite radicals, stabilizing them and giving rise to a relatively stable flavonoids radical [68].

Mechanisms of antioxidant action can include (1) suppression of ROS formation either by inhibition of enzymes or by chelating trace elements involved in free radical generation; (2) scavenging ROS; and (3) upregulation or protection of antioxidant defenses [69, 70]. Flavonoid action involves most of the mechanisms mentioned above. Some of the effects mediated by them may be the combined result of radical scavenging activity and the interaction with enzyme functions. Flavonoids inhibit the enzymes involved in ROS generation, that is, microsomal monooxygenase, glutathione S-transferase, mitochondrial succinoxidase, NADH oxidase, and so forth [71].

**Figure 4:**
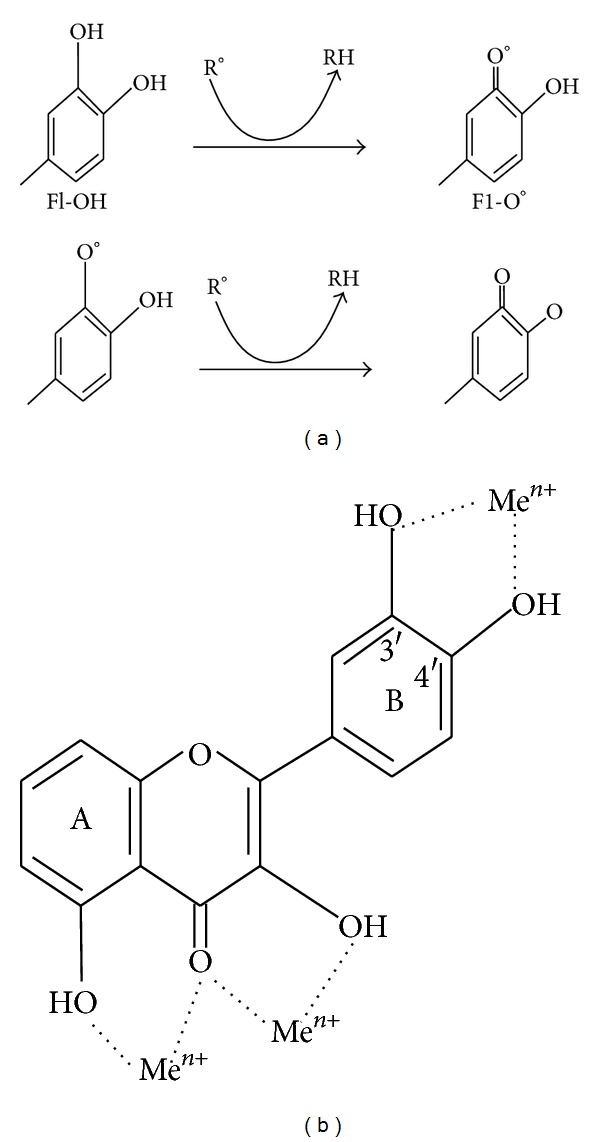
(a) Scavenging of ROS (R°) by flavonoids (Fl-OH) and (b) binding sites for trace metals where Me^*n*+^ indicates metal ions.

Lipid peroxidation is a common consequence of oxidative stress. Flavonoid protect lipids against oxidative damage by various mechanisms [5, 51]. Free metal ions enhance ROS formation by the reduction of hydrogen peroxide with generation of the highly reactive hydroxyl radical. Due to their lower redox potentials flavonoids (Fl-OH) are thermodynamically able to reduce highly oxidizing free radicals (redox potentials in the range 2.13–1.0 V) such as superoxide, peroxyl, alkoxyl, and hydroxyl radicals by hydrogen atom donation (Figure 4(a)). Because of their capacity to chelate metal ions (iron, copper, etc.), flavonoids also inhibit free radical generation [70, 72]. Quercetin in particular is known for its iron-chelating and iron-stabilizing properties. Trace metals bind at specific positions of different rings of flavonoid structures [73]. The binding sites are shown in Figure 4(b).

A 3′,4′-catechol structure in the B ring firmly enhances inhibition of lipid peroxidation. This trait of flavonoids makes them most effective scavengers of peroxyl, superoxide, and peroxynitrite radicals [4]. Epicatechin and rutin are strong radical scavengers and inhibitors of lipid peroxidation *in vitro* [74]. Because of oxidation on the B ring of flavonoids having catechol group a fairly stable orthosemiquinone radical is formed which is strong scavengers. Flavones lacking catechol system on oxidation lead to formation of unstable radicals exhibit weak scavenging potential [75]. The literature shows that flavonoids having an unsaturated 2-3 bond in conjugation with a 4-oxo function are more potent antioxidants than the flavonoids lacking one or both features. Conjugation between the A and B rings allows a resonance effect of the aromatic nucleus that provides stability to the flavonoid radical. Free radical scavenging by flavonoids is potentiated by the presence of both the elements besides other structural features [76].

The flavonoid heterocycle contributes to antioxidant activity by permitting conjugation between the aromatic rings and the presence of a free 3-OH. Removal of a 3-OH annuls coplanarity and conjugation which compromises scavenging ability [77]. It is proposed that B ring OH groups form hydrogen bonds with the 3-OH, aligning the B ring with the heterocycle and A ring. Due to this intramolecular hydrogen bonding, the influence of a 3-OH is enhanced by the presence of a 3′,4′-catechol, elucidating the potent antioxidant activity of flavan-3-ols and flavon-3-ols that possess the latter feature. Generally O-methylation of hydroxyl groups of flavonoids decreases their radical scavenging capacity [76].

Occurrence, position, structure, and total number of sugar moieties in flavonoid (flavonoids glycosides) play an important role in antioxidant activity. Aglycones are more potent antioxidants than their corresponding glycosides. There are reports that the antioxidant properties of flavonol glycosides from tea declined as the number of glycosidic moieties increased [78]. Though glycosides are usually weaker antioxidants than aglycones, bioavailability is sometimes enhanced by a glucose moiety. In the diet, flavonoid glycosidic moieties occur most frequently at the 3- or 7-position [79]. Increasing degree of polymerization enhances the effectiveness of procyanidins against a variety of radical species. Procyanidin dimers and trimers are more effective than monomeric flavonoids against superoxide anion. Tetramers exhibit greater activity against peroxynitrite and superoxide mediated oxidation than trimers, while heptamers and hexamers demonstrate significantly greater superoxide scavenging properties than trimers and tetramers [80].

### 5.2. Hepatoprotective Activity

Several flavonoids such as catechin, apigenin, quercetin, naringenin, rutin, and venoruton are reported for their hapatoprotective activities [81]. Different chronic diseases such as diabetes may lead to development of hepatic clinical manifestations. glutamate-cysteine ligase catalytic subunit (Gclc) expression, glutathione, and ROS levels are reported to be decreased in liver of diabetic mice. Anthocyanins have drawn increasing attention because of their preventive effect against various diseases. Zhu et al. [82] demonstrated that anthocyanin cyanidin-3-O-*β*-glucoside (C3G) increases hepatic Gclc expression by increasing cAMP levels to activate protein kinase A (PKA), which in turn upregulates cAMP response element binding protein (CREB) phosphorylation to promote CREB-DNA binding and increase Gclc transcription. Increased Gclc expression results in a decrease in hepatic ROS levels and proapoptotic signaling. Furthermore, C3G treatment lowers hepatic lipid peroxidation, inhibits the release of proinflammatory cytokines, and protects against the development of hepatic steatosis [82].

Silymarin is a flavonoids having three structural components silibinin, silydianine, and silychristine extracted from the seeds and fruit of milk thistle *Silybum marianum* (Compositae). Silymarin has been reported to stimulate enzymatic activity of DNA-dependent RNA polymerase 1 and subsequent biosynthesis of RNA and protein, resulting in DNA biosynthesis and cell proliferation leading to liver regeneration only in damaged livers [83]. Silymarin increases proliferating hepatocytes in response to FB1 (Fumonisin B1, a mycotoxin produced by *Fusarium verticillioides*) induced cell death without modulation of cell proliferation in normal livers. The pharmacological properties of silymarin involve the regulation of cell membrane permeability and integrity, inhibition of leukotriene, ROS scavenging, suppression of NF-*κ*B activity, depression of protein kinases, and collagen production [84]. Silymarin has clinical applications in the treatment of cirrhosis, ischemic injury, and toxic hepatitis induced by various toxins such as acetaminophen, and toxic mushroom [85].

Hepatoprotective activities were observed in flavonoids isolated from *Laggera alata* against carbon-tetrachloride (CCl_4_-) induced injury in primary cultured neonatal rat hepatocytes and in rats with hepatic damage. Flavonoids at a concentration range of 1–100 *μ*g/mL improved cell viability and inhibited cellular leakage of hepatocyte aspartate aminotransferase (AST) and alanine aminotransferase (ALT) caused by CCl_4_ [86]. Similarly in an *in vivo *experiment flavonoids at of 50, 100, and 200 mg/kg oral doses significantly reduced the levels of AST, ALT, total protein, and albumin in serum and the hydroxyproline and sialic acid levels in liver. Histopathological examinations also revealed the improvement in damaged liver with the treatment of flavonoid [86].

Several clinical investigations have shown the efficacy and safety of flavonoids in the treatment of hepatobiliary dysfunction and digestive complaints, such as sensation of fullness, loss of appetite, nausea, and abdominal pain. *Equisetum arvense *flavonoids as well as hirustrin and avicularin isolated from some other sources is reported to provide protection against chemically induced hepatotoxicity in HepG2 cells [87, 88].

### 5.3. Antibacterial Activity

Flavonoids are known to be synthesized by plants in response to microbial infection; thus it should not be surprising that they have been found *in vitro* to be effective antimicrobial substances against a wide array of microorganisms. Flavonoid rich plant extracts from different species have been reported to possess antibacterial activity [70, 72, 89, 90]. Several flavonoids including apigenin, galangin, flavone and flavonol glycosides, isoflavones, flavanones, and chalcones have been shown to possess potent antibacterial activity [91].

Antibacterial flavonoids might be having multiple cellular targets, rather than one specific site of action. One of their molecular actions is to form complex with proteins through nonspecific forces such as hydrogen bonding and hydrophobic effects, as well as by covalent bond formation. Thus, their mode of antimicrobial action may be related to their ability to inactivate microbial adhesins, enzymes, cell envelope transport proteins, and so forth. Lipophilic flavonoids may also disrupt microbial membranes [92, 93].

Catechins, the most reduced form of the C3 unit in flavonoid compounds, have been extensively researched due to their antimicrobial activity. These compounds are reported for their *in vitro* antibacterial activity against *Vibrio cholerae*, *Streptococcus mutans*, *Shigella*, and other bacteria [94, 95]. The catechins have been shown to inactivate cholera toxin in *Vibrio cholera *and inhibit isolated bacterial glucosyltransferases in *S. mutans,* probably due to complexing activities [94, 96]. Robinetin, myricetin, and (−)-epigallocatechin are known to inhibit DNA synthesis in *Proteus vulgaris. *Mori et al. [97] suggested that the B ring of the flavonoids may intercalate or form hydrogen bond with the stacking of nucleic acid bases and further lead to inhibition of DNA and RNA synthesis in bacteria. Another study demonstrated inhibitory activity of quercetin, apigenin, and 3,6,7,3′,4′-pentahydroxyflavone against *Escherichia coli *DNA gyrase [98].

Naringenin and sophoraflavanone G have intensive antibacterial activity against methicilline resistant *Staphylococcus aureus* (MRSA) and streptococci. An alteration of membrane fluidity in hydrophilic and hydrophobic regions may be attributed to this effect which suggests that these flavonoids might reduce the fluidity of outer and inner layers of membranes [99]. The correlation between antibacterial activity and membrane interference supports the theory that flavonoids may demonstrate antibacterial activity by reducing membrane fluidity of bacterial cells. The 5,7-dihydroxylation of the A ring and 2′,4′-or 2′,6′-dihydroxylation of the B ring in the flavanone structure is important for anti-MRSA activity [100]. A hydroxyl group at position 5 in flavanones and flavones is important for their activity against MRSA. Substitution with C8 and C10 chains may also enhance the antistaphylococcal activity of flavonoids belonging to the flavan-3-ol class [101]. Osawa et al. have shown that 5-hydroxyflavanones and 5-hydroxyisoflavanones with one, two, or three additional hydroxyl groups at the 7, 2′ and 4′ positions inhibited the growth of *S. mutans *and *Streptococcus sobrinus* [102].

Haraguchi and colleagues [100] studied antibacterial activity of two flavonoids, licochalcones A and C, isolated from the roots of *Glycyrrhiza inflata* against *S. aureus *and *Micrococcus luteus.* They observed that licochalcone A inhibited incorporation of radioactive precursors into macromolecules (DNA, RNA, and protein). This activity was similar to the mode of action of antibiotics inhibiting respiratory chain, since energy is required for active uptake of various metabolites as well as for biosynthesis of macromolecules. After further studies it was suggested that the inhibition site of these flavonoids was between CoQ and cytochrome *c* in the bacterial respiratory electron transport chain [100]. There are many examples that lend support to the prowess of phytoconstituents derived from edible and medicinal plants as potent antibacterial agents [103–105].

### 5.4. Anti-Inflammatory Activity

Inflammation is a normal biological process in response to tissue injury, microbial pathogen infection, and chemical irritation. Inflammation is initiated by migration of immune cells from blood vessels and release of mediators at the site of damage. This process is followed by recruitment of inflammatory cells, release of ROS, RNS, and proinflammatory cytokines to eliminate foreign pathogens, and repairing injured tissues. In general, normal inflammation is rapid and self-limiting, but aberrant resolution and prolonged inflammation cause various chronic disorders [106].

The immune system can be modified by diet, pharmacologic agents, environmental pollutants, and naturally occurring food chemicals. Certain members of flavonoids significantly affect the function of the immune system and inflammatory cells [107]. A number of flavonoids such as hesperidin, apigenin, luteolin, and quercetin are reported to possess anti-inflammatory and analgesic effects. Flavonoids may affect specifically the function of enzyme systems critically involved in the generation of inflammatory processes, especially tyrosine and serine-threonine protein kinases [108, 109]. The inhibition of kinases is due to the competitive binding of flavonoids with ATP at catalytic sites on the enzymes. These enzymes are involved in signal transduction and cell activation processes involving cells of the immune system. It has been reported that flavonoids are able to inhibit expression of isoforms of inducible nitric oxide synthase, cyclooxygenase, and lipooxygenase, which are responsible for the production of a great amount of nitric oxide, prostanoids, leukotrienes, and other mediators of the inflammatory process such as cytokines, chemokines, or adhesion molecules [110]. Flavonoids also inhibit phosphodiesterases involved in cell activation. Much of the anti-inflammatory effect of flavonoid is on the biosynthesis of protein cytokines that mediate adhesion of circulating leukocytes to sites of injury. Certain flavonoids are potent inhibitors of the production of prostaglandins, a group of powerful proinflammatory signaling molecules [111].

Reversal of the carrageenan induced inflammatory changes has been observed with silymarin treatment. It has been found that quercetin inhibit mitogen stimulated immunoglobulin secretion of IgG, IgM, and IgA isotypes *in vitro* [112]. Several flavonoids are reported to inhibit platelet adhesion, aggregation, and secretion significantly at 1–10 mM concentration [113]. The effect of flavonoid on platelets has been related to the inhibition of arachidonic acid metabolism by carbon monoxide [114]. Alternatively, certain flavonoids are potent inhibitors of cyclic AMP phosphodiesterase, and this may in part explain their ability to inhibit platelet function.

### 5.5. Anticancer Activity

Dietary factors play an important role in the prevention of cancers. Fruits and vegetables having flavonoids have been reported as cancer chemopreventive agents [72, 115]. Consumption of onions and/or apples, two major sources of the flavonol quercetin, is inversely associated with the incidence of cancer of the prostate, lung, stomach, and breast. In addition, moderate wine drinkers also seem to have a lower risk to develop cancer of the lung, endometrium, esophagus, stomach, and colon [116]. The critical relationship of fruit and vegetable intake and cancer prevention has been thoroughly documented. It has been suggested that major public health benefits could be achieved by substantially increasing consumption of these foods [117].

Several mechanisms have been proposed for the effect of flavonoids on the initiation and promotion stages of the carcinogenicity including influences on development and hormonal activities [118]. Major molecular mechanisms of action of flavonoids are given as follows:

1. downregulation of mutant p53 protein,
2. cell cycle arrest,
3. tyrosine kinase inhibition,
4. inhibition of heat shock proteins,
5. estrogen receptor binding capacity,
6. inhibition of expression of Ras proteins.

Mutations of p53 are among the most common genetic abnormalities in human cancers. The inhibition of expression of p53 may lead to arrest the cancer cells in the G2-M phase of the cell cycle. Flavonoids are found to downregulate expression of mutant p53 protein to nearly undetectable levels in human breast cancer cell lines [119]. Tyrosine kinases are a family of proteins located in or near the cell membrane involved in the transduction of growth factor signals to the nucleus. Their expression is thought to be involved in oncogenesis via an ability to override normal regulatory growth control. Drugs inhibiting tyrosine kinase activity are thought to be possible antitumor agents without the cytotoxic side effects seen with conventional chemotherapy. Quercetin was the first tyrosine kinase inhibiting compound tested in a human phase I trial [120]. Heat shock proteins form a complex with mutant p53, which allows tumor cells to bypass normal mechanisms of cell cycle arrest. Heat shock proteins also allow for improved cancer cell survival under different bodily stresses. Flavonoids are known to inhibit production of heat shock proteins in several malignant cell lines, including breast cancer, leukemia, and colon cancer [119].

Recently it has been shown that the flavanol epigallocatechin-3-gallate inhibited fatty acid synthase (FAS) activity and lipogenesis in prostate cancer cells, an effect that is strongly associated with growth arrest and cell death [116, 121]. In contrast to most normal tissues expression of FAS is markedly increased in various human cancers. Upregulation of FAS occurs early in tumor development and is further enhanced in more advanced tumors [122].

Quercetin is known to produce cell cycle arrest in proliferating lymphoid cells. In addition to its antineoplastic activity, quercetin exerted growth-inhibitory effects on several malignant tumor cell lines *in vitro*. These included P-388 leukemia cells, gastric cancer cells (HGC-27, NUGC-2, NKN-7, and MKN-28), colon cancer cells (COLON 320 DM), human breast cancer cells, human squamous and gliosarcoma cells, and ovarian cancer cells [119]. Markaverich et al. [123] proposed that tumor cell growth inhibition by quercetin may be due to its interaction with nuclear type II estrogen binding sites (EBS). It has been experimentally proved that increased signal transduction in human breast cancer cells is markedly reduced by quercetin acting as an antiproliferative agent [124].

Barnes [125] has extensively reviewed the anticancer effects of genistein on *in vitro* and *in vivo* models. In an study to determine effects of isoflavones genistein, daidzein, and biochanin A on mammary carcinogenesis, genistein was found to suppress the development of chemically induced mammary cancer without reproductive or endocrinological toxicities. Neonatal administration of genistein (a flavonoid) exhibited a protective effect against the subsequent development of induced mammary cancer in rats [126]. Hesperidin, a flavanone glycoside, is known to inhibit azoxymethanol induced colon and mammary cancers in rats [127]. The anticancer properties of flavonoids contained in citrus fruits have been reviewed by Carroll et al. [128]. Several flavonols, flavones, flavanones, and the isoflavone biochanin A are reported to have potent antimutagenic activity [129]. A carbonyl function at C-4 of the flavone nucleus was found to be essential for their activity. Flavone-8-acetic acid has also been shown to have antitumor effects [130]. In earlier studies ellagic acid, robinetin, quercetin, and myricetin have been shown to inhibit the tumorigenicity of BP-7, 8-diol-9, and 10-epoxide-2 on mouse skin [131].

Higher consumption of phytoestrogens, including isoflavones and other flavonoids, has been shown to provide protection against prostate cancer risk [132]. It is well known that due to oxidative stress cancer initiation may take place and thus potent antioxidants show potential to combat progression of carcinogenesis. Potential of antioxidant as an anticancer agent depends on its competence as an oxygen radical inactivator and inhibitor [70, 72, 133]. Therefore diets rich in radical scavengers would diminish the cancer-promoting action of some radicals [134].

### 5.6. Antiviral Activity

Natural compounds are an important source for the discovery and the development of novel antiviral drugs because of their availability and expected low side effects. Naturally occurring flavonoids with antiviral activity have been recognized since the 1940s and many reports on the antiviral activity of various flavonoids are available. Search of effective drug against human immunodeficiency virus (HIV) is the need of hour. Most of the work related with antiviral compounds revolves around inhibition of various enzymes associated with the life cycle of viruses. Structure function relationship between flavonoids and their enzyme inhibitory activity has been observed. Gerdin and Srensso [135] demonstrated that flavan-3-o1 was more effective than flavones and flavonones in selective inhibition of HIV-1, HIV-2, and similar immunodeficiency virus infections. Baicalin, a flavonoid isolated from *Scutellaria baicalensis *(Lamieaceae), inhibits HIV-1 infection and replication. Baicalein and other flavonoids such as robustaflavone and hinokiflavone have also been shown to inhibit HIV-1 reverse transcriptase [136]. Another study revealed inhibition of HIV-1 entry into cells expressing CD4 and chemokine coreceptors and antagonism of HIV-1 reverse transcriptase by the flavone *O*-glycoside [137]. Catechins are also known to inhibit DNA polymerases of HIV-1. Flavonoid such as demethylated gardenin A and robinetin are known to inhibit HIV-1 proteinase [136]. It has also been reported that the flavonoids chrysin, acacetin, and apigenin prevent HIV-1 activation via a novel mechanism that probably involves inhibition of viral transcription [138].

Various combinations of flavones and flavonols have been shown to exhibit synergism. Kaempferol and luteolin show synergistic effect against herpes simplex virus (HSV). Synergism has also been reported between flavonoids and other antiviral agents. Quercetin is reported to potentiate the effects of 5-ethyl-2-dioxyuridine and acyclovir against HSV and pseudorabies infection [136]. Studies have displayed that flavonols are more active than flavones against herpes simplex virus type 1 and the activity order was found to be galangin, kaempferol, and quercetin [136].

Zandi et al. [139] studied the antidengue virus properties of quercetin, hesperetin, naringin, and daidzein at different stages of DENV-2 (dengue virus type-2) infection and replication cycle. Quercetin was found to be most effective against DENV-2 in Vero cells. Many flavonoids, namely, dihydroquercetin, dihydrofisetin, leucocyanidin, pelargonidin chloride, and catechin, show activity against several types of virus including HSV, respiratory syncytial virus, polio virus and Sindbis virus [135]. Inhibition of viral polymerase and binding of viral nucleic acid or viral capsid proteins have been proposed as antiviral mechanisms of action [139]. List of some flavonoids and their efficacy against viruses is given in Table 4.

## 6. Role of Flavonoids in Plants

### 6.1. Combating Oxidative Stress

Flavonoids have long been reported as serving multiple functions in plants [140]. Various abiotic and biotic factors helps in the generation of ROS in plants leading to oxidative stress. Flavonoid biosynthesis in plants is almost exclusively enhanced due to oxidative stress. They have capacity to absorb the most energetic solar wavelengths (i.e., UV-B and UV-A), inhibit the generation of ROS, and quench ROS once they are formed [141]. Flavonoids discharged primary UV-B screening functions when early plants moved from water to colonize the land. Extent of antioxidant capacity and ability to absorb UV-wavelengths depends upon the nature of substitution on different rings of flavonoids. Dihydroxy B ring substituted flavonoids have a greater antioxidant capacity, while their monohydroxy B ring substituted counterparts have greater ability to absorb UV-wavelengths [141].

The most reactive hydroxyl groups (7-OH in flavones or the 3-OH in flavonols) in flavonoids are generally glycosylated. Glycosylation increases solubility in the aqueous cellular environment, protects the reactive hydroxyl groups from autooxidation [142], and allows the transport of flavonoids from the endoplasmic reticulum to various cellular compartments and their secretion to the plasma membrane and the cell wall [143]. Recent evidence shows that antioxidant flavonoids are located in the nucleus of mesophyll cells and within centers of ROS generation, that is, the chloroplast. Here they can easily quench H_2_O_2_, hydroxyl radical, and singlet oxygen [141, 144].

Oxidative stress due to an excess of excitation energy in the chloroplast may be aggravated under conditions that limit the diffusion of CO_2_ to the carboxylation sites and the efficiency of carboxylation [141, 145]. The environmental limitations to CO_2_ assimilation rate include drought/salinity, low/high temperature, and nutrient scarcity. Under these conditions the activity of ROS detoxifying enzymes may significantly reduce in the chloroplast [146, 147], which in turn upregulates the biosynthesis of ROS scavenging flavonoids. The reducing functions of flavonoids are of key significance in plants under severe stress conditions. These functional roles are concomitant with the very high concentration of dihydroxy B ring substituted flavonoids [148]. Flavonoids have been suggested as representing a secondary antioxidant defense system in plant tissues exposed to different stresses [141]. Lipid peroxidation is the common consequence of oxidative stress which disrupts the cell membrane integrity. Quercetin 3-*O*-rutinoside (rutin) may interact with the polar head of phospholipids at water lipid interface, enhancing membrane rigidity and consequently protecting membranes from oxidative damage [149].

### 6.2. As Growth Regulator

Flavonoids carry out functional roles of amazing significance in plant-environment interactions. Flavonoids (in the nanomolar range) may regulate auxin movement and catabolism. The ability of flavonoids to create auxin gradients translates into phenotypes with different morphoanatomical features [150]. The control of flavonoids on auxin movement may have immense value in the stress-induced morphogenic responses of plants such as the flight strategy of sessile organisms exposed to unfavorable environments [151]. Species rich in dihydroxy flavonoids exhibit phenotypes with strikingly different morphological traits as compared with those rich in monohydroxy flavonoids [152]. Dwarf bushy phenotypes with few, small, and thick leaves to direct sunlight irradiance are usually present in sunny environments, thus protecting leaves located deep in the canopy from light-induced severe cellular homeostasis perturbations. On the contrary, shaded plants, which are rich in kaempferol and/or apigenin derivatives (having negligible concentrations of quercetin derivatives), have long internodes, and large leaf lamina coupled with reduced leaf thickness [151].

Flavonoids at the plasma membrane are effective inhibitors of PIN (pin formed) and MDR (multidrug resistance) glycoproteins that are involved in the cell to cell movement of auxin. The ability of flavonoids to inhibit the activity of the efflux facilitator PIN and MDR proteins depends on the presence of the catechol group in the B ring of the flavonoid skeleton. In addition flavonoids regulate the activity of IAA-oxidase with markedly different effects based on their chemical structure [153]. Recent evidence of a nuclear location of flavonoids (as well as of enzymes of flavonoid biosynthesis) supports that flavonoids are capable of modulating the activity of proteins involved in cell growth. Flavonoids may therefore act as transcriptional regulators [154, 155].

## 7. Microbial Production of Flavonoids

In response to the low production efficiency from plants and chemical synthesis, research groups have directed their attention to the production of flavonoids in microorganisms using metabolic engineering and synthetic biology [156]. Chemical synthesis of flavonoids requires extreme reaction conditions and toxic chemicals [157]. Because of the rapid development in molecular biology tools and the flooding of genome information from a variety of organisms, combinatorial biosynthesis offers an advantage for production of rare and expensive natural products. It can be used in both simple and complex transformations without the tiresome blocking and deblocking steps that are common in organic synthesis [158]. Several prokaryotes and eukaryotes such as *E. coli*, *Saccharomyces cerevisiae*, *Streptomyces venezuelae*, and *Phellinus igniarius,* a medicinal mushroom, have been used for production of flavonoids [12].

### 7.1. Phenylpropanoid Pathway

In plants naringenin chalcone is the precursor for a large number of flavonoids produced by the phenylpropanoid synthetic pathway. Fermentative production by *E. coli *carrying an artificially assembled phenylpropanoid pathway was the first example to show that a nearly complete biosynthetic pathway in plants was established in a heterologous microorganism for production of flavanones from the amino acid precursors, phenylalanine, and tyrosine [159]. As the first step in the phenylpropanoid pathway in plants, phenylalanine is deaminated to yield cinnamic acid by the action of phenylalanine ammonia-lyase (PAL). Cinnamic acid is hydroxylated by cinnamate-4-hydroxylase (C4H) to *p*-coumaric acid, which is then activated to *p*-coumaroyl-CoA by the action of 4-coumarate: CoA ligase. Chalcone synthase (CHS) catalyzes the stepwise condensation of three acetate units from malonyl-CoA with *p*-coumaroyl-CoA to yield naringenin chalcone. Naringenin chalcone is converted to naringenin by chalcone isomerase (CHI) or nonenzymatically *in vitro *[160].

### 7.2. Enhancement of Flavonoid Production

Combination of promoter and target genes; knockout of related genes; overexpression of malonyl-CoA; and construction of artificial P450 enzymes are the key molecular biology technology procedures used in the heterologous production of flavonoids. Every gene from the phenylpropanoid pathway is cloned in the host under the control of the promoter which often plays an important role in the heterologous expression of secondary metabolites. Several promoters have been used to enhance production of flavonoids according to the need of specific host such as T7, ermE*, and GAL1 promoter [12]. The extremely low concentration of malonyl-CoA in the microbial cell was one of the drawbacks in the microbiological production of flavonoids. Through the coordinated overexpression of acetyl-CoA carboxylase genes from *Photorhabdus luminescens* intracellular malonyl-CoA pool was amplified leading to increased production of flavonoids [161]. Supplication of UDP-glucose is also a key effector in the biosynthesis of flavonoids. It was proved in an experiment in which researchers knocked out the udg gene encoding for UDP-glucose dehydrogenase. This resulted in elimination of endogenous UDP-glucose consumption pathway leading to increase in intracellular concentration of UDP-glucose and as a consequence increment in the production of flavanones and anthocyanins was observed [162].

One of the barriers to the production of flavonoids and their related compounds in microorganisms by means of assembling biosynthetic genes to form an artificial pathway is the difficulty in expression of active, membrane-bound cinnamate-4-hydroxylase [163]. This enzyme is not expressed efficiently in bacteria due to its instability and the lack of its cognate cytochrome P450 reductase in the host. An advantage of flavonoid production in yeasts or fungi is their ability to express functionally active microsomal cytochrome P450 enzymes, which are usually difficult to be expressed in an active form in bacterial cells. There are various microsomal cytochrome P450 enzymes that are involved in the flavonoid biosynthesis pathway [164]. Combining bacterial cells and eukaryotic cells in a pot enabled researchers to generate a wider library of natural and unnatural products than any of the previously reported systems. *De novo* production of the key flavonoid intermediate naringenin has been demonstrated for the first time from glucose using an engineered *S. cerevisiae* strain which led to fourfold higher concentrations than reported in earlier studies on de novo biosynthesis [165, 166].

## 8. Conclusion 

Prevention and cure of diseases using phytochemicals especially flavonoids are well known. Fruits and vegetables are natural sources of flavonoids. Variety of flavonoids found in the nature possesses their own physical, chemical, and physiological properties. Structure function relationship of flavonoids is epitome of major biological activities. Medicinal efficacy of many flavonoids as antibacterial, hepatoprotective, anti-inflammatory, anticancer, and antiviral agents is well established. These substances are more commonly used in the developing countries. Therapeutic use of new compounds must be validated using specific biochemical tests. With the use of genetic modifications, it is now possible to produce flavonoids at large scale. Further achievements will provide newer insights and will certainly lead to a new era of flavonoid based pharmaceutical agents for the treatment of many infectious and degenerative diseases.

## Figures and Tables

**Table 1:** Structure of flavonoids.

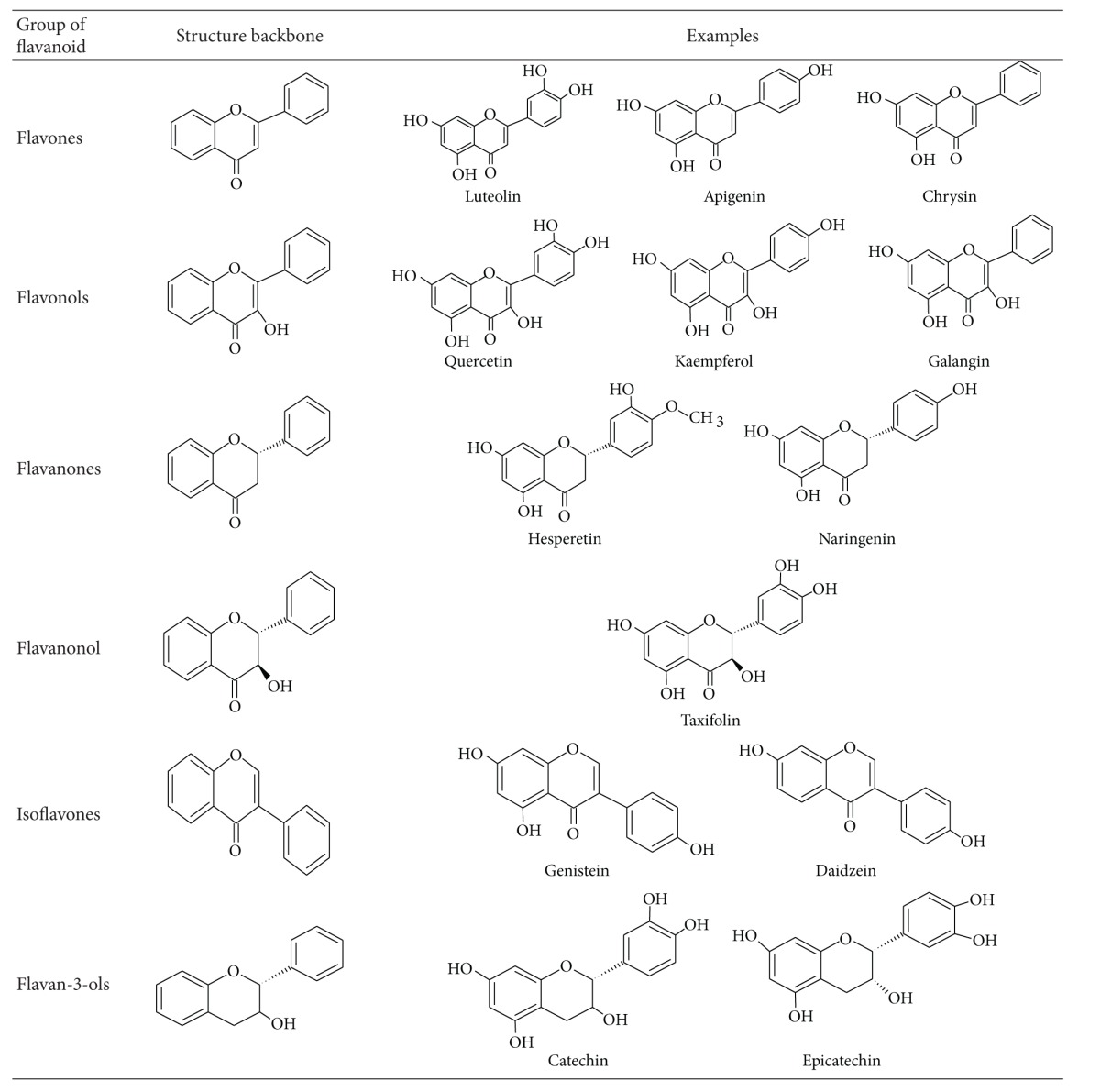

**Table 2:** Classification, structure, and food sources of some dietary flavonoids.

Class	Flavonoid	Dietary source	References
Flavanol	(+)-Catechin (−)-Epicatechin Epigallocatechin	Tea	[14]
Flavone	Chrysin, apigenin Rutin, luteolin, and luteolin glucosides	Fruit skins, red wine, buckwheat, red pepper, and tomato skin	[15–18]
Flavonol	Kaempferol, quercetin, myricetin, and tamarixetin	Onion, red wine, olive oil, berries, and grapefruit.	[17]
Flavanone	Naringin, naringenin, taxifolin, and hesperidin	Citrus fruits, grapefruits, lemons, and oranges	[19, 20]
Isoflavone	Genistin, daidzin	Soyabean	[21]
Anthocyanidin	Apigenidin, cyanidin	Cherry, easberry, and strawberry	[17, 18]

**Table 3:** Medicinal plants rich in flavonoids contents.

Plant	Family	Flavonoid	References
*Aloe vera *	Asphodelaceae	Luteolin	[22]
*Acalypha indica *	Euphorbiaceae	Kaempferol glycosides	[22]
*Azadirachta indica *	Meliaceae	Quercetin	[23]
*Andrographis paniculata *	Acanthaceae	5-hydroxy-7,8-dimethoxyflavone	[24]
*Bacopa moneirra *	Scrophulariaceae	Luteolin	[22]
*Betula pendula *	Betulaceae	Quercetrin	[24]
*Butea monospermea *	Fabaceae	Genistein	[25]
*Bauhinia monandra *	Fabaceae	Quercetin-3-O-rutinoside	[25]
*Brysonima crassa *	Malphigaceae	(+)-catechin	[26]
*Calendula officinalis *	Compositae	isorhamnetin	[24]
*Cannabis sativa *	Compositae	Quercetin	[24]
*Citrus medica *	Rutaceae	hesperidin	[22]
*Clerodendrum phlomidis *	Verbenaceae	Pectolinarigenin,	[23]
*Clitoria ternatea *	Fabaceae	kaempferol-3-neohesperidoside	[27]
*Glyccheriza glabra *	Leguminosae	Liquiritin,	[24]
*Mimosa pudica *	Mimosoideae	Isoquercetin	[28]
*Limnophila indica *	Scrophulariaceae	3,4-methlenedioxyflavone	[28]
*Mentha longifolia *	Lamiaceae	Luteolin-7-O-glycoside	[29]
*Momordica charantia *	Curcurbitaceae	Luteolin	[30]
*Oroxylum indicum *	Bignoniaceaea	Chrysin	[28]
*Passiflora incarnate *	Passifloraceae	Vitexin	[24]
*Pongamia pinnata *	Fabaceae	Pongaflavonol	[31]
*Tephrosia purpurea *	Fabaceae	Purpurin	[28]
*Tilia cordata *	Tiliaceae	hyperoside	[24]

**Table 4:** Antiviral activity of various flavonoids.

Flavonoid	Virus	References
Quercetin	Rabies virus, herpes virus, parainfluenza virus, polio virus, mengo virus, and pseudorabies virus	[32, 33]
Rutin	Parainfluenza virus, influenza virus, and potato virus	[34]
Apigenin	Immunodeficiency virus infection, Herpes simplex virus type, and Auzesky virus	[35]
Naringin	Respiratory syncytial virus	[36]
Luteolin	Auzesky virus	[37]
Morin	Potato virus	[38]
Galangin	Herpes simplex virus type	[39]
